# Endodontic Diagnosis and Nonsurgical Management of a Maxillary Second Molar With Parastyle: Report of a Case With a 24‐Month Follow‐Up

**DOI:** 10.1155/crid/1299580

**Published:** 2026-06-03

**Authors:** Konstantinos Ioannidis, Eirini Sakellaridi, Myron Bitsakis, Nikolaos Economides

**Affiliations:** ^1^ Department of Endodontology, School of Dentistry, Aristoteleio Panepistimio Thessalonikis, Thessaloniki, Greece

**Keywords:** apical periodontitis, cone-beam CT, endodontic treatment, parastyle, root canal anatomy

## Abstract

Parastyle is a rare dental morphological trait characterised by an accessory cusp or tubercle, often found unilaterally on the buccal surface of the mesiobuccal cusp, mainly in the second and third maxillary molars. Its size may range from a small enamel pit to an independent, well‐developed cusp. When clinically present, parastyle may cause complications such as dental caries and sensitivity, localised periodontal disease and occlusal interference. This uncommon anatomical feature may further predispose to diagnostic and treatment challenges when pulp and periapical disease are established. In this case report, a rare second maxillary molar with a parastyle was diagnosed with pulp necrosis and apical periodontitis. Cone‐beam computed tomography was pivotal in addressing the anatomical complexity of the internal root and root canal morphology, particularly regarding the pulp space and the location of the root canal orifices beneath the parastyle. With regard to the efficiency of endodontic management, emphasis was placed on the diagnostic and anatomical challenges posed by such unusual and complex features, as well as on the need for an advanced disinfection regimen. This was addressed with magnification, modern endodontic instrumentation, irrigation and activation techniques, the use of interappointment dressing and root filling with gutta‐percha and a calcium silicate sealer. After 24 months, radiographic follow‐up confirmed a reduction in the size of the periapical lesion, and the tooth was asymptomatic and functional.

## 1. Introduction

Knowledge of dental anatomy is fundamental to successful restorative and endodontic procedures. Substantial inconsistencies in size, number and prevalence of roots and root canals in maxillary molars have been reported in the literature [1]. Maxillary molars may present additional morphological features, including the palatal (P) Carabelli cusp, extra or single roots and c‐shaped configurations [2–4].

The term ‘parastyle’ (PAR) was described a century ago and refers to a type of paramolar cusp or tubercle, expressed unilaterally on the buccal surface of the paracone (mesiobuccal [MB] cusp) of the permanent maxillary molars [5–8]. In rare instances, it develops on the metacone (distobuccal cusp) [5]. An initial theory linked the presence of paramolar tubercles to supernumerary deciduous posterior teeth, which were allegedly fused with adjacent permanent molars during development [6]. However, contemporary researchers are more inclined to conclude that these cusps emerge from the formation of an accessory enamel knot during the morphogenesis [9, 10].

PAR can vary in shape and size, from a simple pit to a well‐developed, lobulated cusp resembling a fused supernumerary tooth [11]. The presence of PAR may cause complications such as dental caries and sensitivity, localised periodontal disease and occlusal interference. The lobulated cusp may contain pulp tissue associated with a root and root canal that may be undeveloped or fully formed and may be interconnected or not to the main pulp chamber [6, 12]. The rarity of PAR renders it ambiguous whether this anatomical disparity is genuinely noteworthy [12]. Cases with a PAR are rare in the literature, and teeth with a PAR requiring root canal treatment are even scarcer [13]. When infected canals are missed, sufficient space and nutrient supply remain to harbour bacteria, resulting in the persistence or progression of periapical disease [14]. Even if missed canals are noninfected, they still function as a potentially vulnerable site for primary infection [14].

The introduction of cone‐beam computed tomography (CBCT) in dentistry has overcome the limitations of periapical radiography, providing three‐dimensional imaging and a high sensitivity and specificity in evaluating hard tissue alterations and additional root and root canal anatomical configurations [15]. The European Society of Endodontology (ESE) has recommended the use of CBCT imaging with a limited field of view (FOV) in patients with extremely complex root canal systems prior to endodontic management based on the ‘as low as reasonably achievable (ALARA)’ principle [16].

Once treatment planning with CBCT is established, using a dental operating microscope becomes essential to improve ergonomics, ensure consistent performance and boost confidence in challenging endodontic cases [17]. The superior illumination and enhanced magnification provided by the microscope help identify complex anatomical structures and navigate narrow or extra canals that might otherwise be overlooked [18]. This results in more predictable and efficient cleaning and removal of infected debris, pulpal tissue remnants and biofilms, particularly in complex anatomical configurations.

The study is aimed at presenting the endodontic management of a complex case involving a maxillary second molar with a PAR, aided by CBCT and magnification with a dental operating microscope.

## 2. Case Description and Results

**Figure 1:**
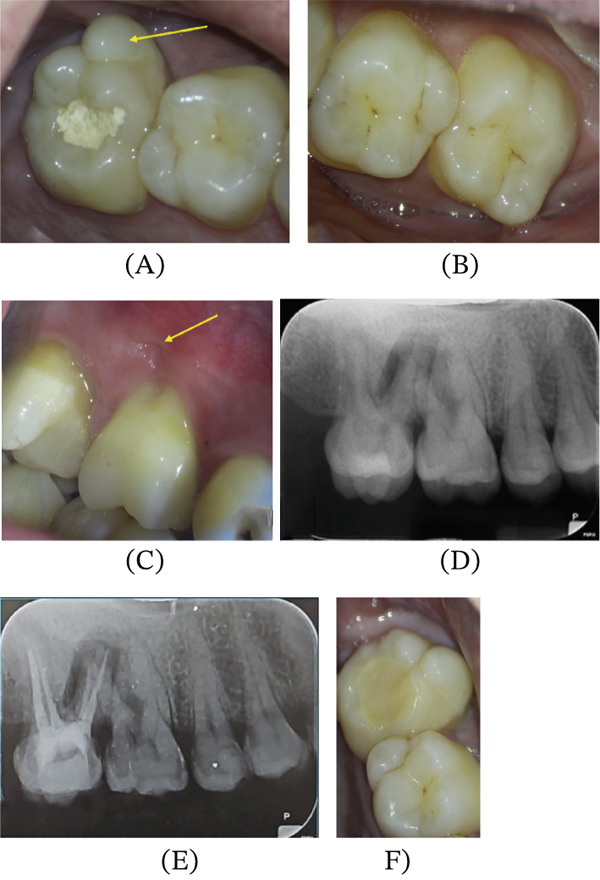
(A) Clinical appearance of parastyle in Tooth #2 (yellow arrow) and its absence in Tooth #3. (B) Clinical appearance of Teeth #14 and #15 (no parastyle present). (C) Active sinus tract in buccal mucosa (yellow arrow). (D) Preoperative radiograph. (E) Postoperative radiograph. (F) Permanent composite core on Tooth #2.

This case report has been written in accordance with the CARE reporting guidelines (https://http://www.care-statement.org/checklist). A 28‐year‐old Caucasian male with a noncontributory medical history was referred to a private endodontic practice for endodontic consultation and treatment of Tooth #2 (right maxillary second molar). According to the referring dentist, an emergency access cavity was initially performed in stages, resulting in negotiation of only the P root canal. Verbal consent was obtained for a full‐mouth examination. Extraoral examination confirmed limited mouth opening and tenderness of the right masseter muscle. Intraorally, the referring dentist confirmed the patient′s periodontal health to be stable (non‐periodontitis). Tooth #2 was tender to percussion and sensitive to mucosal palpation and presented with a cusp‐like bulge on the buccal surface of the MB cusp (Figure 1A). No evident anatomical feature was present on the ipsilateral side of Tooth #15 (Figure 1B). Periodontal examination revealed a narrow 6‐mm periodontal pocket and an active buccal sinus tract (Figure 1C). Sensibility test with ethyl chloride confirmed a negative response from Tooth #2 and a positive response from Tooth #3. The preoperative radiograph confirmed the presence of an endodontic–periodontal lesion associated with Tooth #2, as well as obscure and unusual root canal anatomy (Figure 1D). The initial diagnosis was pulp necrosis and symptomatic apical periodontitis, associated with a Grade 1 endo‐periodontal lesion (EPL) (narrow deep pocket on one tooth surface) in a non‐periodontitis patient [19].

**Figure 2:**
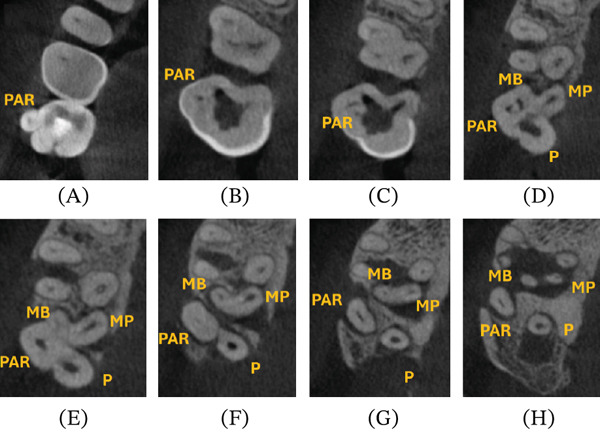
Tooth #2 axial views in CBCT (PAR: parastyle; MB: mesiobuccal; MP: mesiopalatal; P: palatal). (A–C) Clinical crown level. (D) Coronal root third. (E, F) Midroot third, (G, H) Apical root third.

Due to the existing unusual tooth anatomy and the intolerance of the patient to accept the multiple insertions of periapical films, a limited FOV CBCT was indicated (Molars FOV 50X50, automatic selection 84 kV/10 mA, Acteon X‐MIND Prime 3D, de Götzen S.r.l., Varese, Italy). The axial views revealed a complex architecture in the main pulp chamber, divided into four separate compartments, resulting in independent external root morphologies (Figure 2A–H). The paramolar tubercle on the buccal surface was identified as the PAR [13]. A separate PAR root canal was present under the PAR cusp, following a distobuccal diversion towards the apex (Figure 2E–H). The buccal orientation of the PAR resulted in root dehiscence and secondary periodontal involvement (Figure 2F–H). The main mesial root was ribbon‐shaped, splitting into two distinct mesial roots and canals (MB canal and mesiopalatal [MP] canal), remaining interconnected with an isthmus, whilst ending in separate portals of exit apically (Figure 2F–H). The MP canal was located proximal to the P root of Tooth #3. An independent P root and root canal presented with a distopalatal inclination (Figure 2H). According to the CBCT Periapical Index (CBCTPAI), the size and extension of the combined periapical radiolucency, involving the MB, MP and PAR roots, were initially classified as 5D (diameter > 8 mm/destruction of periapical cortical bone) and 4 (4 mm < diameter < 8 mm) for the P root, respectively [20].

**Figure 3:**
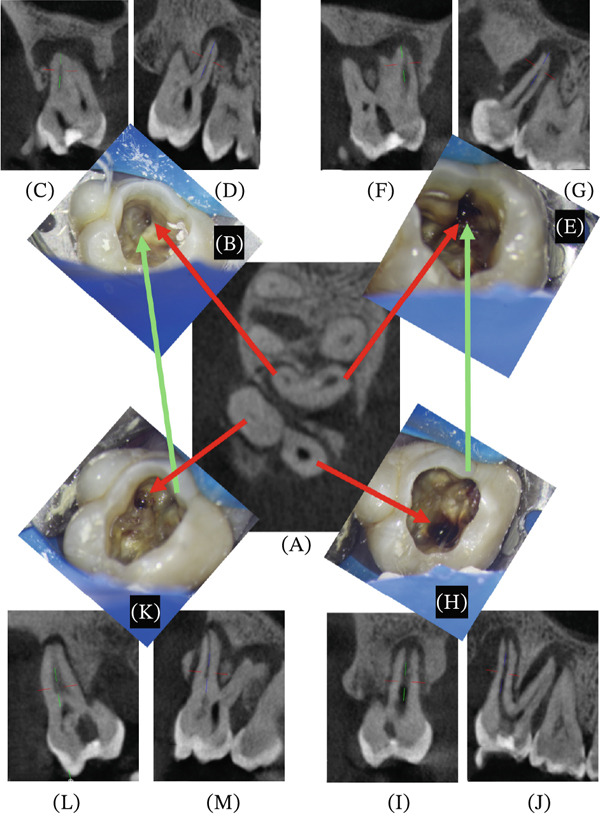
Tooth #2 with parastyle: mapping of internal anatomy of the pulp chamber, in association with the location of the root canal orifices under magnification, an axial CBCT section and centred sagittal and coronal sections of the root canals. (A) Axial CBCT section. (B) MB root canal orifice. (C) CBCT coronal section of the MB root. (D) CBCT sagittal section of the MB root. (E) MP root canal orifice. (F) CBCT coronal section of the MP root. (G) CBCT sagittal section of the MP root. (H) P root canal orifice. (I) CBCT coronal section of the P root. (J) CBCT sagittal section of the P root. (K) Parastyle root canal orifice. (L) CBCT coronal section of the parastyle root. (M) CBCT sagittal section of the parastyle root.

Magnification with dental operating microscope (apochromatic magnichanger 1.6X, objective: f‐250 mm, light source 50 W LED; 100,000 + Lux) (Prima DNT Microscope, Labomed Europe, Capelle aan den IJssel, Netherlands) and centred sagittal and coronal CBCT sections of the root canals aided in the mapping of the internal anatomy of the pulp chamber, in conjunction with the clinical identification of the position of the root canal orifices (13) (Figure 3).

After reviewing the risks, benefits and treatment options with the patient, informed consent was obtained for root canal treatment on #2, which was completed over three visits. The negotiation of root canal orifices was performed using a DG‐16 endodontic probe (GNZ Dental, Madrid, Spain). Mechanical preparation was performed with rotary NiTi files (Protaper Gold, Dentsply Sirona Inc., United Kingdom) in a crown‐down manner, to ensure safe canal negotiation, minimise the risk of iatrogenic errors and appropriately reduce the bacterial and contaminated organic load. Working length determination was performed with K‐file size 20 (Rogin Dental, Shenzhen, China) with the aid of an electronic apex locator (Woodpex V Apex Locator, Woodpecker, Guilin, China). Root canal irrigation was performed with a 27‐G closed‐ended needle (Henry Schein Inc.) and a 5‐mL syringe with a luer‐lock valve (CanalPro Coltene Whaledent, Burgess Hill, United Kingdom). The canals were irrigated with 3% sodium hypochlorite (NaOCl) (Chloracid 3%, Cerkamed, Stalowa Wola, Poland) and 17% ethylenediaminetetraacetic acid (EDTA) (Endo‐Solution, Cerkamed, Stalowa Wola, Poland), as a penultimate rinse. Ultrasonic activation of NaOCl (K25/21, IrriSafe, Acteon) was also performed (3 cycles × 20 s each/per canal), during final rinsing. Pure calcium hydroxide (Cerkamed, Stalowa Wola, Poland) mixed with sterile saline was used as an intracanal medicament between 2‐week appointment intervals. The duration was related to the multistage status of the tooth′s disinfection, as management was extremely complicated due to the need for long sessions, the patient′s limited mouth opening and his ability to attend the surgery. Root canal obturation was performed using a calcium silicate hydraulic sealer (CeraSeal, MetaBiomed Europe GMBH, Mülheim an der Ruhr, Germany) and lateral condensation of gutta‐percha points (Figure 1E). A permanent composite core was added (Figure 1F).

**Figure 4:**
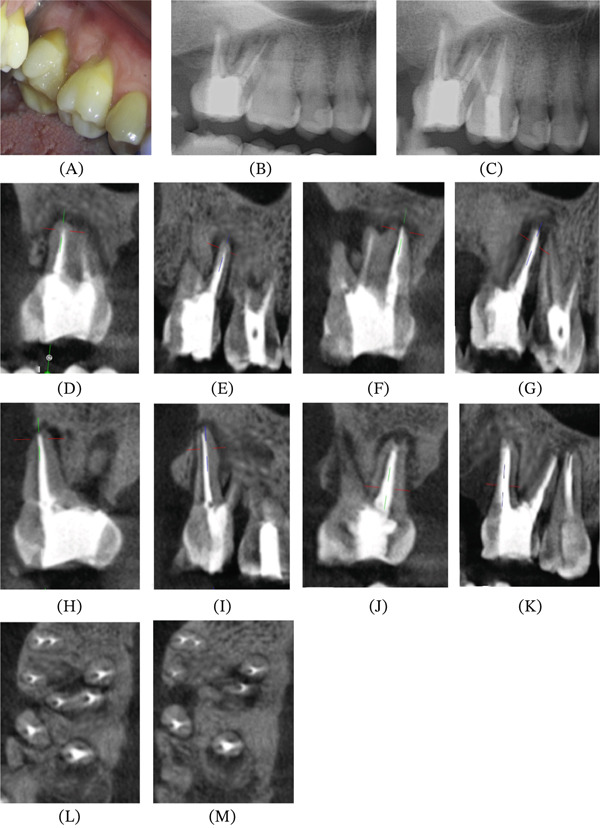
Clinical, radiographic and CBCT follow‐ups. (A) Healing of the mucosal sinus tract in a 12‐month clinical follow‐up. (B) Periapical radiograph after 12 months. (C) Periapical radiograph after 24 months. (D) A 24‐month CBCT coronal section of the MB root. (E) A 24‐month CBCT sagittal section of the MB root. (F) A 24‐month CBCT coronal section of the MP root. (G) A 24‐month CBCT sagittal section of the MP root. (H) A 24‐month CBCT coronal section of parastyle root. (I) A 24‐month CBCT sagittal section of the parastyle root. (J) A 24‐month CBCT coronal section of the P root. (K) A 24‐month CBCT sagittal section of the P root. (L, M) The 24‐month CBCT axial sections in the apical root third.

After 12 months, Tooth #2 was functional and asymptomatic, with soft tissue healing noted (Figure 4A). Radiographic examination was inconclusive, with minor changes in the resolution of the existing lesion on two‐dimensional imaging (Figure 4B). After 24 months, the patient verbally consented to a 24‐month CBCT follow‐up, which confirmed partial healing in the periapical tissues around Tooth #2. The PAR root remained affected by dehiscence (Figure 4D–M). According to the CBCTPAI, the updated periapical status of the MB and MP roots was classified as 3 (diameter of periapical radiolucency > 2 − 4 mm), the updated periapical status of PAR was 3D, and the updated periapical status of the P root was 2 (diameter of periapical radiolucency > 1 − 2 mm), respectively [20]. A sensibility test on Tooth #3 revealed a negative response to ethyl chloride, and a diagnosis of pulp necrosis was made. This finding could be interpreted as a progression of pulpal disease, influenced by localised periodontal involvement and the initial size of the lesion, as shown on the CBCT. These consecutive events may have caused vascular constriction and ischaemia of the blood supply to Tooth #3. To prevent secondary relapse of the periapical tissues, root canal treatment was indicated in response to negative sensibility testing. Hence, Tooth #3 was scheduled for single‐visit root canal treatment and a composite core by the same operator, using a similar root canal treatment protocol (Figure 4C). After 24 months, Teeth #2 and #3 remained functional and asymptomatic, and the patient was satisfied. Periodic recording of periodontal probing depth and tooth survival was recommended.

## 3. Discussion

PAR is a nonmetric trait of particular anatomical interest for palaeontologists, odontologists, dental anthropologists, geneticists and forensic dentists, expressed on the buccal surface of maxillary molars [21, 22]. The available studies associated with the reported prevalence and distribution of PAR in maxillary molars are presented in Table 1. Regarding this trait′s distribution amongst molars, it is most frequently found on the second and third maxillary molars, with its occurrence on the first molar being extremely rare. The prevalence of PAR, according to Kustaloglou, is 0.1% for the first molar, 2.8% for the second molar and 4.7% for the third molar [12]. The percentage of permanent first and second molars with an accessory tubercle on their buccal surfaces was determined by Ooshima et al. to be 0% and 0.9%, respectively [23]. A study by Kazak et al. reported an extremely rare incidence of double PAR on the same two teeth (#17 and #27) [25]. The incidence and expression of PAR may differ in some monozygotic twins. Specifically, of seven instances, only two monozygotic twin pairs showed concordance in PAR occurrence [24]. This indicates that variation in the incidence and expression of PAR is primarily determined by epigenetic effects and/or environmental factors [24].

**Table 1:** Studies reporting the prevalence of parastyle in maxillary first, second and third molars.

Tooth type	Prevalence (%)	Study/author(s) (year)
Maxillary first molar	1/1016 (0.1%)	Kustaloglu (1962) [12]
	0	Ooshima et al. (1996) [23]
	1/1838 dental casts (0.05%)	Scriven et al. (2018) [24]

Maxillary second molar	21/759 (2.8%)	Kustaloglu (1962) [12]
	0.9% in 745 Japanese students	Ooshima et al. (1996) [23]
	17/1838 dental casts (0.9%)	Scriven et al. (2018) [24]

Maxillary third molar	18/379 (4.7%)	Kustaloglu (1962) [12]
	1/1838 dental casts (0.05%)	Scriven et al. (2018) [24]

Due to its apparent low prevalence, there is scarce evidence regarding the prevalence of PAR across populations of different ethnicities [24]. A summary of the prevalence of PAR in maxillary molars, in association with the reported ethnic origin, is presented in Table 2. Kustaloglou examined 293 permanent upper molars of the Southwestern Indian population and found a prevalence of 3%. Compared with other ethnic groups, this percentage was significantly higher [12]. Another study conducted in the South Indian population showed a prevalence of 0.8% [26]. In the reported case, the patient was Caucasian. Due to limited data, it is unsafe to conclude the global prevalence of PAR or the specific racial distribution of this feature in Europe.

**Table 2:** Studies reporting the prevalence of parastyle in different ethnic groups.

Study/author(s) (year)	Population	Sample size and type of examined permanent molars/teeth	Prevalence number/(%)
Kustaloglu (1962) [12]	Southwestern Indians (ad 1100–1600)	293	9/(3.1%)
	White	49	0
	African	64	0
	Melanesian	854	18/(0.02%)
	Filipino	99	0
	Hawaiian	56	0
	Kish (3000 bc)	260	2/(0.8%)
	North Western Indians	335	8/(2.4%)
	Peruvian (ad 900–1400)	144	3/(2.1%)

Ooshima et al. (1996) [23]	Japanese population	1313 first permanent molars	0
		1032 second permanent molars	9/(0.9%)

Kazak et al. (2017) [25]	Turkish population	653 upper and lower molars	11/(0.02%)

Scriven et al. (2018) [24]	Australian Aboriginal	405 dental casts	6/(1.5%)
	Malay Malaysian	293 dental casts	1/(0.3%)
	Chinese Malaysian	196 dental casts	0
	Indian Malaysian	253 dental casts	1/(0.4%)
	Orang Asli	71	1/(1.4%)
	European twins	620	10/(1.6%)

Sureshbabu et al. (2024) [26]	South Indian population	500 extracted teeth	4/(0.8%)

PAR may be associated with a separate root if it is large enough [11]. A study showed that cusps larger than 4 mm in mesiodistal direction were more likely to have their own root canals [27]. In another case of a maxillary left second molar with a large accessory cusp attached to the distobuccal cusp, the main root canal space was shared. Still, their canal orifices were widely apart [28]. The root architecture of three maxillary second molars with PAR attached to their midbuccal surface was examined, using CBCT [29]. In all cases, PARs had their own pulp chamber, their corresponding roots fused externally with the distobuccal root, and root canals merged with the distobuccal canal at various levels [29]. In another report, the PAR root canals merged with both MB and distobuccal root canals, with the aid of CBCT [30]. Likewise, it was found that the PAR root canal may communicate with MB, distobuccal and P root canals [27, 31].

In the present case, a separate root canal existed under the PAR that merged with the distobuccal root. A CBCT scan was required to better understand the tooth′s internal architecture. Periapical radiography remains the primary imaging modality used in routine root canal treatment. Some of its primary drawbacks are the superimposition and the distortion of normal anatomy, since it shows two‐dimensional views of three‐dimensional structures. As we have already established, teeth with PAR are prone to having an extremely complex root canal system. Therefore, the use of periapical radiography appeared to be an inadequate diagnostic feature to disclose the real size, position and root canal layout. Additionally, studies that investigated the prevalence of posttreatment apical periodontitis based on radiographic examinations did not accurately reflect the true proportion of missed canals in all cases due to the limitations of two‐dimensional imaging [32, 33].

Retrospective cross‐sectional studies using CBCT showed that root‐filled teeth with at least one missed canal had a higher prevalence of posttreatment apical periodontitis than teeth with all canals filled. The risk of apical periodontitis was 4.4‐ and 6.25‐fold higher for teeth with at least one untreated canal in the North American and Brazilian populations, respectively [34, 35]. Consequently, CBCT is recommended in cases where PAR is present, as an advanced imaging method to identify all anatomical features of the pulp chamber and the root canal system and to improve treatment outcomes, when combined with the use of a dental operating microscope [16]. The results of a retrospective cohort study showed that microscope‐assisted endodontics in posterior teeth was associated with approximately a threefold increase in positive treatment outcomes compared with endodontic procedures performed without magnification [36].

In our case, after properly shaping all the root canals in a crown‐down manner, an ultrasonic tip was used to activate NaOCl. Using oscillatory motion, ultrasonic tips increase the wall shear stress and consequently propel the irrigant deeper into remote areas of the root canal system, optimising mechanical cleaning [37]. As previously mentioned, teeth displaying a PAR are more likely to have exceedingly complex root canal systems. Consequently, ultrasonic activation enhances the debridement of the root canal system compared to plain syringe irrigation [37]. The interappointment use of calcium hydroxide aided in dissolving any tissue or microbial residues, promoting an alkaline environment, eliminating any potential nutrient source and acting as a physical barrier against potential microleakage [38]. The use of calcium hydroxide is essential in cases involving large periapical lesions to neutralise lipopolysaccharides (LPSs) released by Gram‐negative bacteria during their growth, death or membrane shedding [38]. Additionally, a calcium silicate sealer was applied to leverage its alkalinity, biocompatibility and antimicrobial properties and ability to promote osteogenesis at the apex [39].

The presence of a PAR may have further clinical implications. Between the accessory cusp and the buccal surface of the tooth, a deep groove often forms. This area is highly susceptible to plaque accumulation, which increases the risk of dental caries. These situations require either definitive treatment in accordance with the general principles of caries management or preventive therapy to avoid the onset of caries. In addition, plaque accumulation increases the risk of periodontal disease, such as gingivitis or localised periodontitis [21, 29]. Vertical bone loss may occur along the grooves that divide the cusp from the tooth if they extend to varying depths onto the root surfaces. Oral hygiene management and examinations are strongly advised, even in the absence of signs of periodontal disease [21, 29]. In our case, there was no caries in the PAR groove, but there was a narrow periodontal pocket of 6 mm on the mesial surface of the tooth. Given that the PAR was also located mesiobuccally, it is plausible that this extra cusp contributed to the localised periodontal problem.

In addition to its endodontic relevance, the PAR has clinical significance as it is linked to issues in various dental specialties. Some potential clinical implications include irritation of the buccal mucosa from the sharp, prominent cusp, challenges with bracket cementation and proper alignment of orthodontic archwires, difficulties with clamp adjustment and rubber dam placement and premature tooth contact, which may result in occlusal interference and habitual jaw repositioning. There may also be complications related to tooth preparation for crown restorations and a high risk of pulp exposure due to caries or during restorative procedures [40]. Enameloplasty, which involves selectively removing enamel through grinding, should be reserved for extreme cases, as it can damage a unique variant of dental morphology and potentially harm the pulp beneath the PAR, if present [40]. Since the PAR is located on the buccal side of the affected tooth, it typically does not come into contact with the opposing tooth during occlusion. However, the dentist should remain vigilant for any possible occlusal disturbances or unusual factors.

Last but not least, the existence of PAR is particularly significant for forensic identification. Its rarity enhances its diagnostic importance and clinical value in forensic and dental anthropology, particularly in cases where other identification markers are inadequate [13, 41]. Overall, the implemented clinical approach improved patient‐centred treatment outcomes. The patient′s satisfaction rate was high, as the endodontic treatment planning and management resulted in the retention of functional dentition, periodontal stability and cost‐effectiveness.

## 4. Conclusion

PAR is a rare morphological variation of the human dentition. Conventional radiographs may fail to reveal its complex internal anatomy. Early clinical identification is essential to mitigate the risk of caries and marginal periodontitis, conditions that may compromise pulp status if left undetected. This case demonstrates the pivotal role of CBCT and enhanced magnification and illumination, aided by a dental operating microscope, in diagnosing and managing teeth with PAR.

## Author Contributions

All authors have contributed significantly, and all agree with the manuscript.

## Funding

No funding was received for this manuscript.

## Disclosure

All authors have read and approved the final version of the manuscript. Konstantinos Ioannidis had full access to all of the data in this study and takes complete responsibility for the integrity of the data and the accuracy of the data analysis.

## Conflicts of Interest

The authors declare no conflicts of interest.

## Supporting information


**Supporting Information** Additional supporting information can be found online in the Supporting Information section.  

## Data Availability

The data that support the findings of this study are available on request from the corresponding author. The data are not publicly available due to privacy or ethical restrictions. Supporting Information

## References

[1] Versiani M. A. , Pécora J. D. , and de Sousa-Neto M. D. , Root and Root Canal Morphology of Four-Rooted Maxillary Second Molars: A Micro–Computed Tomography Study, Journal of Endodontics. (2012) 38, no. 7, 977–982, 10.1016/j.joen.2012.03.026, 22703664. 22703664

[2] Ioannidis K. , Lambrianidis T. , Beltes P. , Besi E. , and Malliari M. , Endodontic Management and Cone-Beam Computed Tomography Evaluation of Seven Maxillary and Mandibular Molars With Single Roots and Single Canals in a Patient, Journal of Endodontics. (2011) 37, no. 1, 103–109, 10.1016/j.joen.2010.09.001, 21146087. 21146087

[3] Bhavyaa R. , Sujitha P. , Muthu M. S. , Nirmal L. , and Patil S. S. , Prevalence of the Cusp of Carabelli: A Systematic Review and Meta-Analysis, Annals of Human Biology. (2021) 48, no. 7-8, 572–584, 10.1080/03014460.2022.2032339, 35067147. 35067147

[4] Wahane K. D. , Bansod A. V. , Mattigatti S. , Mahaparale R. , Rote Y. B. , and Wanjari M. B. , Cone-Beam Computed Tomography (CBCT) Analysis of an Unusual Configuration of the Upper First Molar With a C-Shaped Canal With Apically Fused Roots: A Case Report, Cureus. (2023) 15, no. 3, e36474. 37090297 10.7759/cureus.36474 PMC10115750

[5] Scott G. R. T. C. I. , Description and Classification of Permanent Crown and Root Traits, The Anthropology of Modern Human Teeth: Dental Morphology and Its Variation in Recent Human Populations, 1997, 1st edition, Cambridge University Press, 15–69.

[6] Bolk L. , Problems of Human Dentition, American Journal of Anatomy. (1916) 19, no. 1, 91–148, 10.1002/aja.1000190106.

[7] Dahlberg A. A. , The Paramolar Tubercle (Bolk), American Journal of Physical Anthropology. (1945) 3, no. 1, 97–103, 10.1002/ajpa.1330030119.

[8] Haberal M. and Bayraktar Y. , Paramolar Tubercles (Bolk Cusps): Two Case Reports, Journal of Dental Health and Oral Research. (2024) 5, no. 3, 1–6.

[9] Thesleff I. , Developmental Biology and Building a Tooth, Quintessence International. (2003) 34, no. 8, 613–620, 14620213. 14620213

[10] Thesleff I. , Keranen S. , and Jernvall J. , Enamel Knots as Signaling Centers Linking Tooth Morphogenesis and Odontoblast Differentiation, Advances in Dental Research. (2001) 15, no. 1, 14–18, 10.1177/08959374010150010401, 12640732. 12640732

[11] Turner C. G. , Nichol C. R. , and Scott G. R. , Scoring Procedures for Key Morphological Traits of the Permanent Dentition: The Arizona State University Dental Anthropology System, Advances in Dental Anthropology. (1991) 24, 13–31.

[12] Kustaloglu O. A. , Paramolar Structures of the Upper Dentition, Journal of Dental Research. (1962) 41, no. 1, 75–83, 10.1177/00220345620410015001.

[13] Ceinos R. , Bernardi C. , Bertrand M. F. , and Quatrehomme G. , The Parastyle: A Discrete Trait of the Maxillary Molars, Cureus. (2024) 16, no. 9, e69733, 10.7759/cureus.69733, 39429369. 39429369 PMC11490277

[14] Leon-Lopez M. , Montero-Miralles P. , Cabanillas-Balsera D. , Sauco-Marquez J. J. , Martin Gonzalez J. , and Segura-Egea J. J. , Association Between the Presence of Missed Canals, Detected Using CBCT, and Post-Treatment Apical Periodontitis in Root-Filled Teeth: A Systematic Review and Meta-Analysis, Journal of Clinical Medicine. (2025) 14, no. 16, 10.3390/jcm14165781, 40869607. PMC12386526 40869607

[15] Patel K. , Wanf F. M. , Tahmasbi M. , Teixeira F. , Nair M. , and Jalali P. , Detection Thresholds for Root Canal Visibility on High-Resolution CBCT: A Micro-CT Validation Study, Journal of Endodontics. (2026) 52, no. 3, 442–450, 10.1016/j.joen.2025.11.016, 41285308. 41285308

[16] Patel S. , Brown J. , Semper M. , Abella F. , and Mannocci F. , European Society of Endodontology Position Statement: Use of Cone Beam Computed Tomography in Endodontics, International Endodontic Journal. (2019) 52, no. 12, 1675–1678, 10.1111/iej.13187, 31301231. 31301231

[17] Low J. F. , Dom T. N. M. , and Baharin S. A. , Magnification in Endodontics: A Review of Its Application and Acceptance Among Dental Practitioners, European Journal of Dentistry. (2018) 12, no. 4, 610–616, 10.4103/ejd.ejd_248_18, 30369811. 30369811 PMC6178675

[18] Yoshioka T. , Kobayashi C. , and Suda H. , Detection Rate of Root Canal Orifices With a Microscope, Journal of Endodontics. (2002) 28, no. 6, 452–453, 10.1097/00004770-200206000-00008, 12067127. 12067127

[19] Papapanou P. N. , Sanz M. , Buduneli N. , Dietrich T. , Feres M. , Fine D. H. , Flemmig T. F. , Garcia R. , Giannobile W. V. , Graziani F. , and Greenwell H. , Periodontitis: Consensus Report of Workgroup 2 of the 2017 World Workshop on the Classification of Periodontal and Peri-Implant Diseases and Conditions, Journal of Clinical Periodontology.(2018) 45, no. supplement. 20, s162–s170. 29926490 10.1111/jcpe.12946

[20] Estrela C. , Bueno M. R. , Azevedo B. C. , Azevedo J. R. , and Pecora J. D. , A New Periapical Index Based on Cone Beam Computed Tomography, Journal of Endodontics. (2008) 34, no. 11, 1325–1331, 10.1016/j.joen.2008.08.013, 18928840. 18928840

[21] Nabeel S. , Danish G. , Hedge U. , and Gehlot P. M. , Parastyle: Clinical Significance and Management of Two Cases, International Journal of Oral and Maxilofacial Pathology. (2012) 3, no. 2, 61–64.

[22] Turner R. A. and Harris E. F. , Maxillary Second Premolars With Paramolar Tubercles, Dental Anthropology Journal. (2018) 17, no. 3, 75–78, 10.26575/daj.v17i3.150.

[23] Ooshima T. , Ishida R. , Mishima K. , and Sobue S. , The Prevalence of Developmental Anomalies of Teeth and Their Association With Tooth Size in the Primary and Permanent Dentitions of 1650 Japanese Children, International Journal of Paediatric Dentistry. (1996) 6, no. 2, 87–94, 10.1111/j.1365-263X.1996.tb00218.x, 8957846. 8957846

[24] Scriven G. , Roger J. , Brook A. , Scott G. R. , Mihailidis S. , Bin Khamis M. F. , and Townsend G. , Frequency of Occurrence and Degree of Expression of the Parastyle in Several Modern Human Populations, Dental Anthropoly Journal. (2018) 31, no. 1, 3–9.

[25] Kazak M. , Colakoglu G. , Elcin M. A. , Somturk E. , and Gunal S. , Examination of Paramolar Tubercles in Turkish Population Using Cone Beam Computed Tomography, International Journal of Morphology. (2017) 35, no. 4, 1416–1421, 10.4067/S0717-95022017000401416.

[26] Sureshbabu S. , Ramadoss R. , Arthanari A. , and Ramalingam K. , Dental Anomalies: An Identification Marker in Forensics, Cureus. (2024) 16, no. 5, e59922), 10.7759/cureus.59922. 38854347 PMC11157986

[27] Colakoglu G. , Kaya Buyukbayram I. , Elcin M. A. , Kazak M. , and Sezer H. , Evaluation of the Internal Anatomy of Paramolar Tubercles Using Cone-Beam Computed Tomography, Surgical Radiology and Anatomy. (2020) 42, no. 1, 15–21, 10.1007/s00276-019-02361-1, 31659406. 31659406

[28] Thompson B. H. , Endodontic Therapy of an Unusual Maxillary Second Molar, Journal of Endodontics. (1988) 14, no. 3, 143–146, 10.1016/S0099-2399(88)80216-4. 3268631

[29] Ohishi K. , Ohishi M. , Takahashi A. , Kido J. , Uemura S. , and Nagata T. , Examination of the Roots of Paramolar Tuberoles With Computed Tomography: Report of 3 Cases, Oral Surgery Oral Medicine Oral Pathology Oral Radiology and Endodontology. (1999) 88, no. 4, 479–483, 10.1016/S1079-2104(99)70066-1, 10519759. 10519759

[30] Jain P. , Ananthnarayan K. , Ballal S. , and Natanasabapathy V. , Endodontic Management of Maxillary Second Molars Fused With Paramolar Tubercles Diagnosed by Cone Beam Computed Tomography - Two Case Reports, Journal of Dentistry. (2014) 11, no. 6, 726–732, 25628705. 25628705 PMC4281197

[31] Garg S. , Mishra N. , Goel A. , Tikku A. P. , and Bharti R. , Endodontic Management of a Rare Anamoly of Paramolar Tubercle Fused With Maxillary Second Molar Using CBCT-A Case Report, Journal of Dental and Medical Sciences. (2018) 17, no. 7, 52–55.

[32] Georgopoulou M. K. , Spanaki-Voreadi A. P. , Pantazis N. , Kontakiotis E. G. , and Morfis A. S. , Periapical Status and Quality of Root Canal Fillings and Coronal Restorations in a Greek Population, Quintessence International. (2008) 39, no. 2, e85–e92, 18560646. 18560646

[33] Moreno J. O. , Alves F. R. , Goncalves L. S. , Martinez A. M. , Rocas I. N. , and Siqueira J. F. , Periradicular Status and Quality of Root Canal Fillings and Coronal Restorations in an Urban Colombian Population, Journal of Endodontics. (2013) 39, no. 5, 600–604, 10.1016/j.joen.2012.12.020, 23611376. 23611376

[34] Karabucak B. , Bunes A. , Chehoud C. , Kohli M. R. , and Setzer F. , Prevalence of Apical Periodontitis in Endodontically Treated Premolars and Molars With Untreated Canal: A Cone-Beam Computed Tomography Study, Journal of Endodontics. (2016) 42, no. 4, 538–541, 10.1016/j.joen.2015.12.026, 26873567. 26873567

[35] Costa F. F. N. P. , Pacheco-Yanes J. , Siqueira J. F. , Oliveira A. C. S. , Gazzaneo I. , Amorim C. A. , Santos P. H. B. , and Alves F. R. F. , Association Between Missed Canals and Apical Periodontitis, International Endodontic Journal. (2019) 52, no. 4, 400–406, 10.1111/iej.13022. 30284719

[36] Chang Y. C. and Wang T. Y. , Effectiveness of Microscope-Assisted Root Canal Treatment in Permanent Posterior Teeth: A Retrospective Cohort Study, Journal of Dentistry. (2025) 157, 105771, 10.1016/j.jdent.2025.105771, 40268114. 40268114

[37] Căpută P. E. , Retsas A. , Kuijk L. , de Paz L. E. C. , and Boutsioukis C. , Ultrasonic Irrigant Activation During Root Canal Treatment: A Systematic Review, Journal of Endodontics. (2019) 45, no. 1, 31–44.e13, 10.1016/j.joen.2018.09.010. 30558797

[38] Ordinola-Zapata R. , Noblett W. C. , Perez-Ron A. , Ye Z. , and Vera J. , Present Status and Future Directions of Intracanal Medicaments, International Endodontic Journal. (2022) 55, no. supplement 3, 613–636, 10.1111/iej.13731, 35322427. PMC9321724 35322427

[39] Cardinali F. and Camilleri J. , A Critical Review of the Material Properties Guiding the Clinician′s Choice of Root Canal Sealers, Clinical Oral Investigations.(2023) 27, no. 8, 4147–4155, 10.1007/s00784-023-05140-w, 37460901. 37460901 PMC10415471

[40] Paras Mull J. and Manjunath M. K. , Paramolar Tubercle in Endodontics: An Overview, Case Report and Specimen Study, Journal of Pierre Fauchard Academy. (2013) 27, no. 4, 124–128, 10.1016/j.jpfa.2013.12.004.

[41] Carneiro J. L. , Santos A. , Magalhães T. , Afonso A. , and Caldas I. M. , Human Identification Using Dental Techniques A Case Report, Medical Science and Law. (2015) 55, no. 2, 78–81, 10.1177/0025802414531752. 24757022

