# Supplementary material for: Endodontic Diagnosis and Nonsurgical Management of a Maxillary Second Molar With Parastyle: Report of a Case With a 24‐Month Follow‐Up
Source: Case Rep Dent. 2026 Jun 3;2026:1299580. doi: 10.1155/crid/1299580 (PMC13239487; doi:10.1155/crid/1299580)
Supplement: Supplementary file 1 — Supporting Information Additional supporting information can be found online in the Supporting Information section. [file CRID-2026-1299580-s001.docx]

***All the patients allowed personal data processing and informed consent was obtained from all individual participants included in the study"***
